# Few-shot cross-episode adaptive memory for metal surface defect semantic segmentation

**DOI:** 10.1038/s41598-026-36445-x

**Published:** 2026-01-18

**Authors:** Jiyan Zhang, Hanze Ding, Ming Peng, Shuzhen Tu, Guiping Chen, Yanfang Liu

**Affiliations:** 1 College of Mathematics and Information Engineering, Longyan University, Longyan, 364012 China; 2 Longyan Tobacco Industrial Co. Ltd., Longyan, 364000 China

**Keywords:** Few-shot semantic segmentation, Metal surface defect, Cross-episode semantic dependence, Global response mask average pooling, Attention distillation, Computational biology and bioinformatics, Mathematics and computing

## Abstract

Few-shot semantic segmentation has gained significant attention in metal surface defect detection due to its ability to segment unseen object classes with only a few annotated defect samples. Previous methods constrained to single-episode training suffer from limited adaptability in semantic description of defect regions and coarse segmentation granularity. In this paper, we propose an episode-adaptive memory network (EAMNet) that specifically addresses subtle variances between episodes during training. The episode adaptive memory unit (EAMU) leverages an adaptive factor to model semantic dependencies across different episodes. The context adaptation module (CAM) aggregates hierarchical features of support-query pairs for fine-grained segmentation. The proposed global response mask average pooling (GRMAP) introduces a global response normalization to obtain fine-grained cues directly from the support prototype. We also introduce an attention distillation (AD), which leverages fine-grained semantic attention correspondence to process defect region cues and stabilize the cross-episode adaptation in EAMU. Extensive experiments demonstrate that our approach establishes new state-of-the-art performance on both Surface Defect-$4^i$ and FSSD-12 datasets.

## Introduction

Metal surface defect detection^1–3^ is an essential quality control process on assembly lines. Different from conventional classification^4–7^ and anchor box detection methods^8^, semantic segmentation attracted extensive attention recently for its specific location description^9,10^. However, the generalization capability of most metal surface defect segmentation networks is constrained by their dependency on large annotated datasets and poor transferability to unseen classes. Data collection and manual annotation consume substantial time and resources, particularly for dense prediction tasks.

**Fig. 1:**
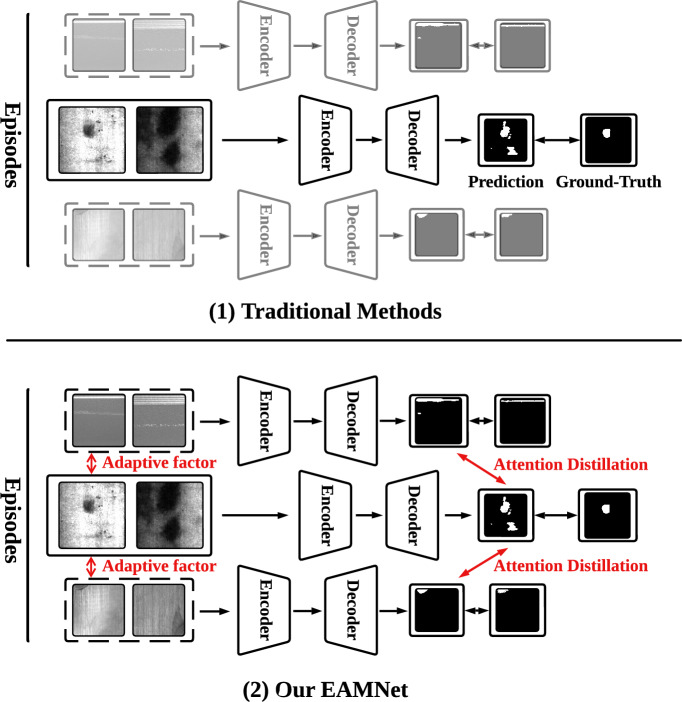
Comparison between (1) Traditional methods and (2) Our EAMNet. (1) Traditional methods extract semantic knowledge from a single episode, which is not able to learn the holistic semantic patterns. (2) Our EAMNet utilizes an adaptive factor and an attention distillation to capture the semantic knowledge across episodes.

Few-shot semantic segmentation (FSS)^11,12^ emerges as a promising approach to address these challenges. Specifically, FSS is formulated as a series of episodes, each containing a support set and a query set. In contrast to supervised semantic segmentation^13^, which only segments the same classes presented in the training set, FSS utilizes a few annotated support samples to segment unseen classes in the query set. Therefore, the core of FSS centers on how to best utilize the information in the support set. However, the limited support knowledge available in a single episode compromises the model’s adaptability to various defect types and often yields coarse segmentation results. As illustrated in Fig. 1(1), traditional methods typically conduct feature extraction and fusion operations confined within a single episode to generate pixel-level mask. When combined with well-designed feature processing, these methods can deliver competitive performance. Nevertheless, the form of single episode hinders the models from learning the category distribution and semantic characteristics of the support set, thus limiting their ability to fully capture the defect patterns. This constraint fundamentally causes both limitations by inducing overfitting to episodic features like the brightness of a scratch. Consequently, models lack semantic adaptability and produce coarse segmentation, as they cannot discern essential defect characteristics.

In this paper, we propose an episode adaptive memory network (EAMNet) to overcome the above mentioned drawbacks. Specifically, as shown in Fig. 1(2), we design an episode adaptive memory unit (EAMU) to explore the relations between episodes and conduct the cross-episode interaction. The EAMU generates an adaptive factor to model the semantic dependencies across episodes for defect regions. Further, we propose context adaption module (CAM) and global response mask average pooling (GRMAP) to segment fine-grained defect regions. Considering the effectiveness of deep level features in capturing category distribution and semantic patterns, CAM employs the deep level features of the support-query pair within a single episode to enable fine-grained prediction. Simultaneously, GRMAP further enhance the effectiveness of the support prototype through regulating the state of mask average pooling via global response normalization^14^. The limited discriminability of early-stage features during training poses a significant challenge to these modules, particularly the EAMU, which relies on high-quality multi-level features. To overcome this bottleneck, we present an attention distillation (AD). The AD exploits attention correspondence at fine-grained resolutions, meticulously extracting and transferring key semantic information across soft-target from attention map. This mechanism empowers the model to analyze the semantic cues across episodes rapidly and adapt to diverse scenarios effectively.

Our contributions can be summarized as follows:

1. An EAMU generates a cross-episode adaptive factor to exploit the semantic dependence across episodes for metal surface defect regions.
2. We propose a CAM and a GRMAP leveraging hierarchical features and global response normalization respectively to accomplish fine-grained segmentation.
3. To accelerate cross-episode semantic learning and enhance generalization, we introduce an AD that transfers fine-grained semantic attention from high-resolution features.
4. Our EMANet sets new state-of-the-art results on both standard benchmarks: Surface Defect-$4^i$ and FSSD-12.

## Related work

### Metal surface defect detection

In metal surface defect detection, image classification, object detection, and semantic segmentation represent the fundamental computer vision approaches. Notably, semantic segmentation-based methodologies, which offer pixel-level precision in prediction outcomes, have recently garnered significant attention. Chao et al.^15^ conduct an information augmentation and multiscale feature fusion algorithm based on YOLOv8, which uses the information augmentation network to reduce the information loss during feature downsampling extraction. Several studies employ unsupervised learning methods to address metal surface defect challenges^16,17^. Meanwhile, alternative approaches leverage attention mechanisms^18,19^ to generate defect attention maps for enhanced localization. Zhang et al.^20^ construct the innovative triple-attention mechanism to enhance the models’ ability of expressing defect characteristics. Wei et al.^21^ introduce a vision transformer model that combines receptive-field attention convolution and context broadcasting median to handle the high variability and sample imbalance in metal defects. Zhang et al.^22^ design a dual-branch local-guided global self-attention network to achieve sufficient attention in local details. Jin et al.^23^ construct a human guidance to address the data scarcity and effectively leverage expert knowledge. Zhao et al.^24^ propose a cross-supervised contrastive learning domain adaptation network with transformer to solve the differences that steel defects exhibit in appearance and background. The aforementioned detection methods fail to address the critical issue of labeled sample scarcity in metal defect inspection. The unsupervised and semi-supervised approaches offer partial remedies but struggle with reliability. To directly overcome these limitations, our EAMNet introduces a few-shot learning paradigm, which systematically addresses sample scarcity through meta-learning and achieves reliable generalization via explicit prototype-based learning within each episode.

### Few-shot semantic segmentation

Few-shot semantic segmentation (FSS) generates dense masks for novel classes using minimal annotated support samples. Extending the prototypical learning framework from^25^, contemporary FSS approaches^26,27^ construct class-specific prototypes to capture discriminative feature representations. Yang et al.^28^ propose a bi-orientated rectification few-shot segmentation network based on fine-grained prototypes to address the limitations of current methods that only extract general target prototype. Chen et al.^29^ conduct a mask generation module and an iterative refinement module, respectively addressing the inherent two challenges of locating segmented objects and deriving class-specific features in the absence of support mask and semantic labels. Wang et al.^30^ leverage semantic word embedding and query set self-supplementing information to handle the inter-class interference and information loss of generalized few-shot semantic segmentation. While these prototype-based methods remain confined to a single episode, they overlook the valuable semantic information across episodes. To overcome the single-episode limitation of these methods, EAMNet employs an EAMU to model cross-episode correlations, which then enables its CAM, GRMAP, and AD to perform fine-grained segmentation.

### Knowledge distillation

Knowledge distillation (KD)^31^ is a widely-adopted technique for model compression. Larger models typically exhibit greater representational capacity, while compact models feature fewer parameters, higher computational efficiency, and lower deployment costs. The core objective of KD is to transfer knowledge from a high-capacity teacher model to a lightweight student model while maintaining acceptable performance levels. The approach in ^32^ implements a self-distillation framework to perform internal knowledge transfer which can enhance the model accuracy without external supervision. Self-distillation operates by partitioning the model into multiple components and facilitating the transfer of knowledge from deeper layers to shallower ones. Additionally, self-distillation is employed to exploit the intrinsic semantic correlations of models. Li et al.^33^ utilize self-distillation from the last batch during model training to maximize the performance. Lu et al.^34^ introduce a multi-stage dynamic anchor decoder that aggregation capabilities of the sparse attention mechanism to improve the effectiveness of self-distillation. Peng et al.^35^ leverage the hierarchical attention maps to create self-distillation resolution. Motivated by^34,35^, we design an attention distillation conducting the self-distillation of attention maps and ground-truth to enhance the adaptability of our model.

## Method

### Problem setting

Our implementation adopts the standard few-shot segmentation paradigm^36,37^ based on episodic meta-learning. We partition the dataset into training set $D_\textrm{train}$ and testing set $D_\textrm{test}$, each containing numerous randomly sampled episodes. Each episode comprises a support set $\mathscr {S}$ and query set $\mathscr {Q}$ from the same class *c*. During meta-training, models learn on classes $C_{\textrm{train}}$ and are evaluated on disjoint unseen classes $C_{\textrm{test}}$ ($C_{\textrm{train}} \cap C_{\textrm{test}} = \varnothing$). The FSS model processes $\mathscr {S} = \left\{ \left( I_S^i, M_S^i \right) \right\} _{i=1}^K$ and $\mathscr {Q} = \left\{ \left( I_Q, M_Q \right) \right\}$ from class $c \in C_{\textrm{train}}$ to predict query mask $\hat{\boldsymbol{M}}_Q$, where $I_S^i, I_Q \in \mathbb {R}^{3 \times H \times W}$ are RGB images. And $\boldsymbol{M}_S^i, \boldsymbol{M}_Q \in \{0,1\}^{H \times W}$ are binary masks, where $H \times W$ denotes spatial dimensions. During training, both $\boldsymbol{M}_Q$ and $\boldsymbol{M}_S$ are utilized. At test time, only $\boldsymbol{M}_S$ is available as input. Crucially, the meta-trained model directly generalizes to novel categories without test-time optimization, as it leverages support semantic cues to localize query regions of interest.

**Fig. 2:**
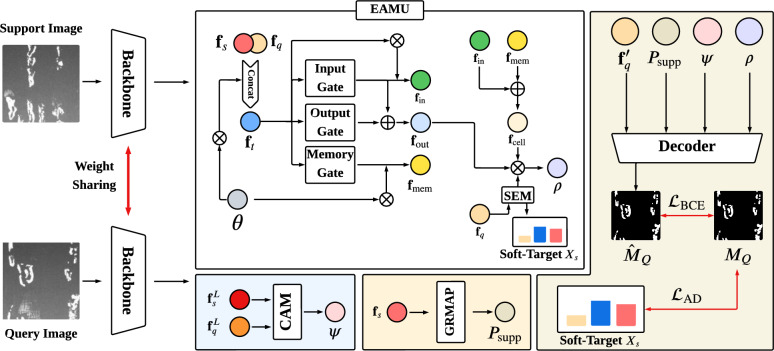
Overview of our episode adaptive memory network with the EAMU, CAM, GRMAP and AD. where $\otimes$ denotes matrix multiplication and $\oplus$ represents matrix addition.

### Overview

As illustrated in Fig. 2, our Episode Adaptive Memory Network (EAMNet) comprises four core components: episode adaptive memory unit (EAMU), context adaptive module (CAM), global response mask average pooling (GRMAP), and attention distillation (AD). More precisely, for the support image $I_S$ and query image $I_Q$, we employ a shared backbone network to extract *L*-level features concurrently, where $L=4$. Subsequently, we introduce the EAMU, which is designed to generate an adaptive factor $\rho$ within the given support-query image pair. This factor, which captures and retains transferable meta-knowledge across different tasks, operates at the cross-episode level Meanwhile, high-resolution features are processed by CAM, which aims to produce context adaptive factor $\psi$ for support-query pairs through capturing category distribution and semantic patterns. CAM functions within a single episode, specializing in fine-grained and spatial context alignment between the current support and query features. To achieve precise prediction, we then employ the GRMAP, which learns to generate the prototype $\boldsymbol{P}_\textrm{supp}$ from support foreground feature by the global response normalization. GRMAP acts as a support enhancer prior to the query adaptation process and focus on producing a robust and globally-aware prototype. We then feed the $\rho$, $\psi$, $\boldsymbol{P}_\textrm{supp}$ and the query feature $\textbf{f}_q$ filtered by the activation function into a decoder to predict the final segmentation mask $\hat{\boldsymbol{M}}_Q$ for the query image. Then the model can be trained under the supervision of a binary cross-entropy loss $\mathscr {L}_\textrm{BCE}$ computed between the ground-truth mask $\boldsymbol{M}_Q$ and the predicted mask $\hat{\boldsymbol{M}}_Q$. Moreover, we design an extra AD in the end of training stage to enhance the semantic expression at fine-grained resolutions by $\mathscr {L}_\textrm{AD}$. It is crucial to note that AD is an optimization-oriented module distinct from the inference path, designed exclusively to accelerate convergence through knowledge distillation across network stages. Next, we provide a detailed elaboration of each of the previously mentioned modules.

### Episode adaptive memory unit

Inspired by SG-One^38^, recent FSS models for metal surface defect^39,40^ usually leverage the prototypes from the support and query set within a single episode to generate the prediction mask. As the constrained knowledge provided within a single episode, the cross-episode semantic description of defect regions for prototypes is not incorporated into the model. To counter this, the EAMU is employed to derive a cross-episode adaptive factor from the cumulative support-query pairs across episodes by gate units^41^, enabling the model to adaptively remember inter-episode semantic dependencies. Different from sequence-modeling memories (e.g., LSTM^41^) or static global memories (e.g., Transformer memory^42^), our EAMU achieves an episode-level memory for meta-learning. Designed for meta-learning, our EAMU operates at an episodic granularity, maintaining a dynamic memory seed that updates only upon episodic task completion. Consequently, the gate units of EAMU consolidate cross-episode experience and guide adaptation, thereby explicitly addressing inter-episode distribution shifts rather than modeling sequences or storing global knowledge. As illustrated in Fig. 2, the EAMU receives as input the current episode’s support-query feature pair $\textbf{f}_s, \textbf{f}_q \in \mathbb {R}^{c \times h \times w}$ where *c*, *h* and *w* denotes the channels, heights and width of the features respectively. Note that the support-query feature pair $\textbf{f}_s$ and $\textbf{f}_q$ are generated by the feature pyramid network^43^, which conduct the first three level feature maps to obtain the contextual information. Then EAMU employs an architecture comprising an input gate $\mathscr {F}_\textrm{in}$, output gate $\mathscr {F}_\textrm{out}$, and memory gate $\mathscr {F}_\textrm{mem}$ to adaptively retain the semantic details within the current episode, where the $\mathscr {F}_\textrm{in}$, $\mathscr {F}_\textrm{out}$ and $\mathscr {F}_\textrm{mem}$ are comprised of various convolution and activation functions. To perform the cross-episode knowledge, the input gate $\mathscr {F}_\textrm{in}$ combines the current episode features $\textbf{f}_t \in \mathbb {R}^{2c \times h \times w}$ and the memory seed $\theta \in \mathbb {R}^{h \times w}$. It outputs a condensed feature $\textbf{f}_\textrm{in} \in \mathbb {R}^{1 \times h \times w}$ that encapsulates the novel semantic information learned from the current episode:

1 $$\begin{aligned} & \begin{aligned} \textbf{f}_t=\mathscr {C} \left( \textbf{f}_q, \textbf{f}_s \right) \otimes \theta , \end{aligned} \end{aligned}$$

2 $$\begin{aligned} & \begin{aligned} \textbf{f}_\textrm{in}= \mathscr {F}_\textrm{in} \left( \textbf{f}_t \right) \otimes \textbf{f}_t, \end{aligned} \end{aligned}$$

where $\mathscr {C}$ is the concatenation operation, $\otimes$ represents matrix multiplication and $\theta$ is a trainable parameter. Then, the output of semantic information $\textbf{f}_\textrm{out} \in \mathbb {R}^{1 \times h \times w}$ in the current episode is processed by output gate $\mathscr {F}_\textrm{out}$:

3 $$\begin{aligned} \begin{aligned} \textbf{f}_\textrm{out}=\mathscr {F}_\textrm{out} \left( \textbf{f}_t \right) \oplus \mathscr {F}_\textrm{in} \left( \textbf{f}_t \right) , \end{aligned} \end{aligned}$$

where the $\oplus$ denotes matrix addition.

Having the memory seed $\theta$, we further process the cross-episode semantic description through memory gate $\mathscr {F}_\textrm{mem}$:

4 $$\begin{aligned} \begin{aligned} \textbf{f}_\textrm{mem}=\mathscr {F}_\textrm{mem} \left( \textbf{f}_t \right) \otimes \theta , \end{aligned} \end{aligned}$$

where $\textbf{f}_\textrm{tmem} \in \mathbb {R}^{1 \times h \times w}$ initializes the memory information of defect regions, and $\textbf{f}_\textrm{mem} \in \mathbb {R}^{1 \times h \times w}$ leverages $\theta$ to obtain the holistic cross-episode semantic description. To achieve the corss-episode interaction, we integrate the new feature $\textbf{f}_\textrm{in}$ with the cross-episode semantic description $\textbf{f}_\textrm{mem}$ to generate the cell information $\textbf{f}_\textrm{cell} \in \mathbb {R}^{1 \times h \times w}$:

5 $$\begin{aligned} \begin{aligned} \textbf{f}_\textrm{cell}= \textbf{f}_\textrm{in} \oplus \textbf{f}_\textrm{mem}. \end{aligned} \end{aligned}$$

Next, EAMNet utilizes a squeeze & excitation module (SEM)^44^ to further process the fine-grained semantic information from query feature in current episode:

6 $$\begin{aligned} \begin{aligned} \rho = \textbf{f}_\textrm{cell} \otimes \textbf{f}_\textrm{out} \otimes \mathscr {F}_\textrm{SEM}\left( \textbf{f}_q \right) , \end{aligned} \end{aligned}$$

where $\mathscr {F}_\textrm{SEM}$ is the SEM, $\rho \in \mathbb {R}^{1 \times h \times w}$ denotes the adaptive factor which contains cross-episode semantic details and cues.

### Context adaptive module

Given the feature maps of $L=4$ level processed by backbone, i.e., $\left\{ \textbf{f}_q^l \right\} _{l=1}^L \in \mathbb {R}^{lc \times \frac{h}{2^l+2} \times \frac{w}{2^l+2}}$ and $\left\{ \textbf{f}_s^l \right\} _{l=1}^L \in \mathbb {R}^{lc \times \frac{h}{2^l+2} \times \frac{w}{2^l+2}}$, the contextual category distribution and semantic patterns can be captured through deep level feature maps. Specifically, we calculate the cosine similarity $\boldsymbol{D}_\textrm{cos}^{l} \in \mathbb {R}^{1 \times \frac{h}{2^l+2} \times \frac{w}{2^l+2}}$ of last two-stage support-query pairs $\left\{ \textbf{f}_q^l \right\} _{l=L-1}^L$ and $\left\{ \textbf{f}_s^l \right\} _{l=L-1}^L$ for fine-grained semantic description, i.e., $\boldsymbol{D}_\textrm{cos}^{L}$ and $\boldsymbol{D}_\textrm{cos}^{L-1}$. As well as the procedure for multiple stage pairs follows analogously:

7 $$\begin{aligned} \begin{aligned} \boldsymbol{D}_\textrm{cos}^{L}= \mathscr {F}_\textrm{SIM}\left( \textbf{f}_q^L, \textbf{f}_s^L \otimes \mathscr {R}_\textrm{mask} \left( \boldsymbol{M}_S \right) \right) , \end{aligned} \end{aligned}$$

where $\mathscr {F}_\textrm{SIM}$ calculates the cosine similarity of support-query pair, $\mathscr {R}_\textrm{mask}$ reshapes the support mask $\boldsymbol{M}_S$ to be the same shape as $\textbf{f}_s^\textrm{high}$. Then we integrate the $\boldsymbol{D}_\textrm{cos}^{L}$ and $\boldsymbol{D}_\textrm{cos}^{L-1}$ to generate the context adaptive factor $\psi \in \mathbb {R}^{2 \times h \times w}$ which depicts the contextual semantic pattern of the defect regions in current episode:

8 $$\begin{aligned} \begin{aligned} \psi = \mathscr {R}_\textrm{factor}\left( \lambda \left( \mathscr {C} \left( \boldsymbol{D}_\textrm{cos}^{L}, \mathscr {R}_\textrm{cos} \left( \boldsymbol{D}_\textrm{cos}^{L-1} \right) \right) \right) \otimes \beta \right) , \end{aligned} \end{aligned}$$

where $\lambda$ is an activation function, $\beta$ controls the adaptive weight of $\psi$ during downstream and is initialized to 0.4, $\mathscr {R}_\textrm{cos}$ reshapes the $\boldsymbol{D}_\textrm{cos}^{L-1}$ to be the same shape as $\boldsymbol{D}_\textrm{cos}^{L}$ and $\mathscr {R}_\textrm{factor}$ reshapes the input shape to be the $\mathbb {R}^{2 \times h \times w}$.

### Global response mask average pooling

Following the general form^35,38^, the key of mask average pooling is the knowledge of support set. However, current methods that take support features as input directly suffer from coarse-grained foreground knowledge. Thus, we introduce the global response normalization^14^ to enhance the effectiveness of support knowledge. Specifically, given the *c*-channel support feature $\textbf{f}_s=\left\{ \textbf{z}_1, \textbf{z}_2,...,\textbf{z}_i \right\} _{i=1}^{c}$, we employ a response normalization to compute the feature normalization scores $\mathscr {N} \in \mathbb {R}^{c \times 1 \times 1}$:

9 $$\begin{aligned} \begin{aligned} \mathscr {N}=\frac{{\Vert \textbf{z}_i \Vert }_2}{{\sum _{j=1}^c\Vert \textbf{z}_j \Vert }_2}, \end{aligned} \end{aligned}$$

where ${\Vert \textbf{z}_i \Vert }_2$ is the L2-norm of the *i*-th channel. Subsequently, we calibrate the original input features based on $\mathscr {N}$ to generate the responsive support feature $\textbf{f}_s^\prime \in \mathbb {R}^{c \times h \times w}$:

10 $$\begin{aligned} \begin{aligned} \textbf{f}_s^\prime =\left( \textbf{f}_s \otimes \eta \mathscr {N}\right) + \tau , \end{aligned} \end{aligned}$$

where $\eta$ and $\tau$ are learnable parameters. For holistic semantic information, we also produce the responsive query feature $\textbf{f}_q^\prime \in \mathbb {R}^{c \times h \times w}$ through Eqs. (9)–(10) in the downstream work.

Then we apply the mask average pooling to capture the guide prototype $\boldsymbol{P}_\textrm{supp} \in \mathbb {R}^{c \times h \times w}$ from $\textbf{f}_s^\prime$:

11 $$\begin{aligned} \begin{aligned} \boldsymbol{P}_\textrm{supp} = \mathscr {F}_\textrm{AVG} \left( \textbf{f}_s^\prime \otimes \boldsymbol{M}_S \right) , \end{aligned} \end{aligned}$$

where $\mathscr {F}_\textrm{AVG}$ is a 2D average pooling operation.

The enhanced query feature $\textbf{f}_q^\prime$, guide prototype $\boldsymbol{P}_\textrm{supp}$, context adaptive factor $\psi$ and cross-episode adaptive factor $\rho$ are all reshape to the same spatial size and concatenated to a representative prototype $\boldsymbol{P}_\textrm{final} \in \mathbb {R}^{\left( 2c + 3 \right) \times h \times w}$:

12 $$\begin{aligned} \begin{aligned} \boldsymbol{P}_\textrm{final} = \mathscr {C} \left( \textbf{f}_q^\prime , \boldsymbol{P}_\textrm{supp}, \psi , \rho \right) . \end{aligned} \end{aligned}$$

Finally, $\boldsymbol{P}_\textrm{final}$ is fed into a decoder to produce segmentation mask $\hat{\boldsymbol{M}_Q}$:

13 $$\begin{aligned} \begin{aligned} \hat{\boldsymbol{M}}_Q = \mathscr {F}_\textrm{cls} \left( \mathscr {F}_\textrm{conv} \left( \boldsymbol{P}_\textrm{final} \right) \right) , \end{aligned} \end{aligned}$$

where $\mathscr {F}_\textrm{conv}$ and $\mathscr {F}_\textrm{cls}$ are two consecutive modules that constitute the decoder.

### Attention distillation

The construction of the cross-episode adaptive factor relies on features from all network stages. However, in early training, the shallow layers yield coarse-grained features due to limited supervision, which slows model convergence and degrades the quality of factor. To address this, we introduce an attention distillation, which refines these early-stage features by transferring knowledge from deeper layers, thereby preserving fine-grained information without compromising cross-episode learning. Moreover, compared to the one-hot labels in ground-truth annotations, the soft targets used in distillation capture inter-category dependencies, thereby providing richer semantic details within individual episodes. Specifically, we apply the $\mathscr {F}_\textrm{SEM}$ to collect the soft-target $\boldsymbol{X}_s$ of query feature $\textbf{f}_q$:

14 $$\begin{aligned} \begin{aligned} \boldsymbol{X}_s=\mathscr {F}_\textrm{softmax} \left( \mathscr {F}_\textrm{SEM} \left( \textbf{f}_q \right) \right) , \end{aligned} \end{aligned}$$

where $\mathscr {F}_\textrm{softmax}$ is a softmax layer. Then the KL (Kullback-Leibler) divergence loss is used as supervision from the teacher to student with their softmax output:

15 $$\begin{aligned} \begin{aligned} \mathscr {L}_\textrm{AD} = \boldsymbol{X}_t \textrm{log} \left( \boldsymbol{X}_t \right) - \boldsymbol{X}_t\textrm{log} \left( \boldsymbol{X}_s \right) , \end{aligned} \end{aligned}$$

where $\boldsymbol{X}_t$ is the ground-truth $\boldsymbol{M}_Q$.

Finally, the loss function of our model during training can be formulated as:

16 $$\begin{aligned} \begin{aligned} \mathscr {L} = \mathscr {L}_\textrm{BCE} + \alpha \mathscr {L}_\textrm{AD}, \end{aligned} \end{aligned}$$

where $\alpha$ is a hyperparameter which set to 0.05.

### Extend to *K*-shot settings

Thus far, our discussion has focused on the one-shot setting, as summarized in Fig. 2. To extend EAMNet to the *K*-shot scenario (where *K* support images per category are available), we leverage the per-episode adaptive factor. To preserve spatial information and maintain consistency across varying shot counts, we directly concatenate the support features $\textbf{f}_s^l = \mathscr {C} \left( \textbf{f}_s^{l,1}, \textbf{f}_s^{l,2},..., \textbf{f}_s^{l,K} \right)$ along the channel dimension, along with their corresponding masks.

## Experiments

### Datasets

Following the setting of^37,45,46^, we use metal surface defect datasets, i.e., Surface Defect-$4^i$^37^ and FSSD-12^45^, to evaluate EAMNet. Surface Defect-$4^i$ is a general dataset containing 12 various classes of metal surface defect. FSSD-12 is a strip steel surface defect dataset and also contains 12 categories.

For each dataset, we perform 3-fold cross-validation by partitioning all classes equally. Following^37,45^, we maintain identical class splits for Surface Defect-$4^i$ and FSSD-12. Two folds serve as training data, with the remaining fold reserved for testing.

### Metric and evaluation

Following established practice, we adopt mean Intersection-over-Union (mIoU) and foreground-background IoU (FB-IoU) as evaluation metrics. For testing, we follow the protocol from^47^ to ensure experimental validity. Specifically, each experiment consists of five independent trials using distinct random seeds, with final results representing the average across these trials.

### Implementation details

EAMNet is implemented in PyTorch and trained episodically for 200 epochs on Surface Defect-$4^i$ and FSSD-12. All models are trained on 4 NVIDIA GeForce RTX 3090 GPUs with batch size 2, and tested on a single GPU with batch size 1. The optimizer employed is consistent with^37^, and the learning rate is set to 0.0001. To validate our method’s backbone-agnostic efficacy, we evaluate it with ResNet-50^48^ and VGG-16^49^ backbone. During inference, predictions are resized to match original input resolution while preserving ground-truth labels.

### Baseline

First, we exclude the EAMU, CAM, GRMAP, and AD from EAMNet. Then, we replace the enhanced query feature $\textbf{f}_q^\prime$ in the downstream network with the original query feature $\textbf{f}_q$ to construct the baseline model. The remaining experimental settings are kept consistent with those of EAMNet.

### Comparison with state-of-the-art methods

#### Surface defect-$4^i$

Table 1 compares mIoU performance on the Surface Defect-$4^i$ dataset between our method and several representative models. The results demonstrate that: (1) EAMNet achieves state-of-the-art (SOTA) performance under both 1-shot and 5-shot settings. Notably, when using the VGG-16 backbone, EAMNet surpasses MAPTNet^46^ (the previous SOTA) by significant margins of $4.43\%$ and $5.89\%$ for 1-shot and 5-shot, respectively, which demonstrates that the effective cross-episode adaptation and intra-episode feature processing of EAMNet are key to its superior performance. (2) EAMNet substantially outperforms the baseline. For instance, with the VGG-16 backbone, EAMNet achieves $35.13\%$ mIoU compared to the baseline’s $23.08\%$. This improvement stems from the synergistic effect of its core components, with EAMU handling cross-episode adaptation, CAM managing intra-episode refinement, GRMAP responsible for support enhancement, and AD ensuring stable convergence.

EAMNet achieves competitive efficiency with 54.47G FLOPs, indicative of its lightweight and adaptable design for diverse tasks. A detailed comparison of computational costs with TGRNet, CPANet, and MAPTNet is presented in Table 2.

**Table 1:** Comparison of EAMNet with state-of-the-art methods and semantic networks for metal surface defect FSS on Surface Defect-$4^{i}$, evaluated by mIoU and FB-IoU under 1-shot and 5-shot settings using the VGG-16 and ResNet-50 backbone. The **best** and second best results are highlighted accordingly. Improv. (%) represents the percentage improvement in mIoU over the *Baseline.*.

Methods	Backbone	1-shot						5-shot					
		Fold-0	Fold-1	Fold-2	Mean	FBIoU	Improv. (%)	Fold-0	Fold-1	Fold-2	Mean	FBIoU	Improv. (%)
HDMNet^35^		31.10	28.91	21.26	27.09	52.76	$\uparrow$4.01	42.33	28.00	25.97	32.10	51.45	$\uparrow$4.47
DCPNet^36^	VGG16	28.68	27.45	24.08	26.74	52.22	$\uparrow$3.65	22.91	27.81	25.59	25.44	51.96	$\downarrow$2.20
TGRNet(1-normal)^37^		29.78	25.15	24.36	26.43	51.50	$\uparrow$3.35	37.42	24.66	26.52	29.53	53.27	$\uparrow$1.90
CPANet^45^		22.03	25.05	24.07	23.72	51.35	$\uparrow$0.63	30.11	25.95	19.26	25.11	52.21	$\downarrow$2.53
MAPTNet^46^		39.88	25.98	**26.24**	30.70	50.76	$\underline{\uparrow 7.62}$	39.93	31.66	29.82	33.80	56.33	$\underline{\uparrow 6.17}$
PFENet^47^		23.28	19.45	20.48	21.07	51.14	$\downarrow$2.01	27.94	21.67	25.24	24.95	53.99	$\downarrow$2.68
*Baseline*		*28.33*	*24.54*	*16.38*	*23.08*	*49.12*	–	*38.11*	*24.87*	*19.92*	*27.63*	*53.67*	-
EAMNet		**47.01**	**32.91**	25.46	**35.13**	**54.57**	$\uparrow$**12.04**	**54.90**	**32.64**	**31.53**	**39.69**	**58.28**	$\uparrow$**12.06**
HDMNet^35^		35.58	**40.79**	27.50	34.62	56.01	$\uparrow$8.41	38.62	41.11	**32.61**	37.45	56.19	$\uparrow$9.76
DCPNet^36^	ResNet50	27.19	31.96	24.68	27.94	51.67	$\uparrow$1.73	42.78	39.35	32.21	38.11	58.77	$\underline{\uparrow 10.43}$
TGRNet(1-normal)^37^		35.46	32.37	24.75	30.86	53.62	$\uparrow$4.65	41.61	28.66	27.87	32.71	53.00	$\uparrow$5.03
CPANet^45^		32.52	29.65	24.66	28.94	51.94	$\uparrow$2.73	39.36	37.84	27.82	35.01	57.73	$\uparrow$7.32
MAPTNet^46^		41.27	40.20	22.78	34.75	55.61	$\underline{\uparrow 8.54}$	46.49	40.85	26.20	37.85	58.22	$\uparrow$10.16
PFENet^47^		29.45	24.90	16.21	23.52	54.06	$\downarrow$2.69	33.98	30.07	22.78	28.94	56.92	$\uparrow$1.26
HMNet^50^		39.49	28.10	23.73	30.44	53.97	$\uparrow$4.23	44.00	30.19	27.33	33.84	56.74	$\uparrow$6.15
*Baseline*		*34.84*	*24.17*	*19.63*	*26.21*	*49.42*	–	*34.55*	*24.53*	*23.98*	*27.69*	*50.66*	–
EAMNet		**44.47**	40.39	**28.38**	**37.75**	**59.25**	$\uparrow$**11.53**	**51.85**	**41.25**	31.76	**41.62**	**59.23**	$\uparrow$**13.93**

**Table 2:** Comparison with metal surface defect FSS in computational cost on Surface Defect-$4^i$. The **best** results are highlighted accordingly.

Methods	mIoU	FLOPs	#Params.
TGRNet	26.43	83.69G	**9.38M**
CPANet	23.72	162.23G	11.98M
MAPTNet	30.70	66.80G	16.80G
EAMNet	**35.13**	**54.47G**	15.79M

#### FSSD-12

FSSD-12 is an extra validation dataset for the generalization of our model which only contains strip steel surface defect samples. Table 3 compares mIoU and FB-IoU performance on the FSSD-12 dataset. Our EAMNet significantly outperforms recent methods in both 1-shot and 5-shot settings using either VGG-16 or ResNet-50 backbones. With ResNet-50, EAMNet achieves mIoU improvements of $1.92\%$ over MAPTNet^46^ (1-shot) and $0.73\%$ over HDMNet^35^ (5-shot) by leveraging episode adaptation to exploit latent data correlations for enhanced training and prediction. In addition, EAMNet gains significant improvement over the baseline models. For example, EAMNet with VGG-16 backbone achieves $15.93\%$ and $15.02\%$ mIoU improvement over the baseline model, which proves the superiority of our model in such challenging scenarios.

**Table 3:** Comparison of EAMNet with state-of-the-art methods and semantic networks for metal surface defect FSS on FSSD-12, evaluated by mIoU and FB-IoU under 1-shot and 5-shot settings using the VGG-16 and ResNet-50 backbone. The **best** and second best results are highlighted accordingly. Improv. (%) represents the percentage improvement in mIoU over the *Baseline.*.

Methods	Backbone	1-shot						5-shot					
		Fold-0	Fold-1	Fold-2	Mean	FBIoU	Improv. (%)	Fold-0	Fold-1	Fold-2	Mean	FBIoU	Improv. (%)
HDMNet^35^		50.12	49.05	45.51	48.23	66.57	$\uparrow$6.10	48.26	49.60	46.11	47.99	66.69	$\uparrow$3.10
DCPNet^36^	VGG16	53.30	44.52	40.98	46.27	66.01	$\uparrow$4.14	50.07	48.69	43.07	47.28	67.28	$\uparrow$2.39
TGRNet(0-normal)^37^		63.74	51.68	49.95	55.12	73.36	$\uparrow$13.00	**66.16**	60.24	51.07	59.16	74.48	${\underline{\uparrow 14.27}}$
CPANet^45^		50.90	47.39	53.38	50.56	69.26	$\uparrow$8.43	50.15	37.41	43.39	43.65	64.73	$\downarrow$1.24
MAPTNet^46^		**65.87**	55.59	51.49	57.65	72.90	${\underline{\uparrow 15.52}}$	62.84	58.93	47.41	56.39	74.88	$\uparrow$11.50
PFENet^47^		43.65	37.89	36.12	39.22	67.72	$\downarrow$2.91	44.86	40.66	36.57	40.70	68.92	$\downarrow$4.19
*Baseline*		48.40	*38.04*	*39.94*	*42.13*	*63.94*	-	*48.85*	*43.98*	*41.84*	*44.89*	*65.24*	–
EAMNet		63.75	**56.00**	**54.43**	**58.06**	**74.46**	$\uparrow$**15.93**	65.73	**61.87**	**52.14**	**59.91**	**75.68**	$\uparrow$**15.02**
HDMNet^35^		60.50	**65.46**	51.42	59.13	74.33	$\uparrow$12.33	63.03	**68.22**	53.74	61.66	77.33	${\underline{\uparrow 11.78}}$
DCPNet^36^	ResNet50	59.65	63.13	51.84	58.21	74.79	$\uparrow$11.41	61.68	61.80	52.71	58.73	74.10	$\uparrow$8.85
TGRNet(0-normal)^37^		61.09	63.24	51.28	58.54	75.20	$\uparrow$11.74	61.59	65.81	56.27	61.22	76.74	$\uparrow$11.34
CPANet^45^		54.40	52.59	48.39	51.79	65.15	$\uparrow$4.99	56.73	55.06	51.92	54.57	71.19	$\uparrow$4.69
MAPTNet^46^		68.20	58.24	**55.34**	60.59	75.64	${\underline{\uparrow 13.79}}$	63.57	62.52	52.85	59.65	73.47	$\uparrow$9.76
PFENet^47^		49.00	47.87	41.78	46.22	73.76	$\downarrow$0.58	50.11	50.98	42.34	47.81	74.77	$\downarrow$2.07
HMNet^50^		62.15	54.96	50.56	55.89	71.10	$\uparrow$9.09	60.21	60.02	50.53	56.92	72.12	$\uparrow$7.04
*Baseline*		*53.95*	*43.53*	*42.92*	*46.80*	*64.66*	–	*55.84*	*50.24*	*43.57*	*49.88*	*67.74*	-
EAMNet		**68.73**	64.11	54.71	**62.52**	**75.80**	$\uparrow$**15.72**	**64.35**	65.92	**56.91**	**62.39**	**78.24**	$\uparrow$**12.51**

#### Qualitative results

**Fig. 3:**
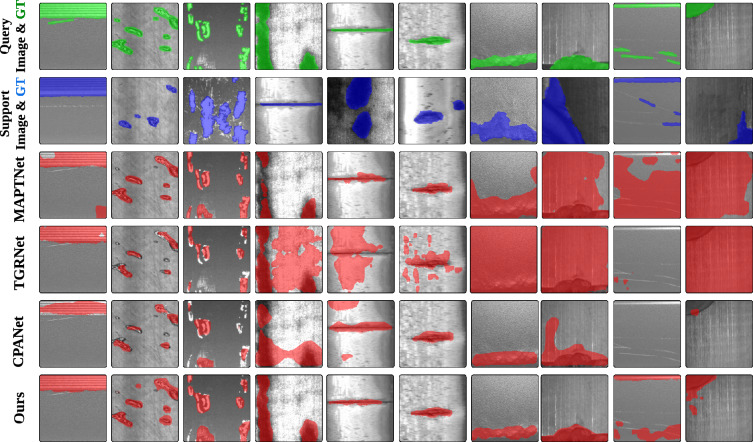
Qualitative results of the MAPTNet, TGRNet, CPANet and proposed EAMNet on Surface Defect-$4^i$.

We report some qualitative results generated from several models for metal surface defect detection and our EAMNet on the Surface Defect-$4^i$ benchmarks. Compared with these representative models, EAMNet exhibits the following advantages as shown in Fig. 3.

1. EAMNet can more accurately segment the target class, while the previous methods incorrectly segments the seen classes as the target classes (1st to 3rd columns).
2. EAMNet can capture subtle defect details for semantic description of defect regions to address the limited adaptability problem caused by single-episode (4th to 6th columns).
3. EAMNet can provide better fine-grained resolution through the contextual adaptive factor from CAM and the support guidance from GRMAP (7th to 8th columns).
4. Some failure case are exhibited from 9th to 10th columns. These challenges stems from inherent problem in the data, where the model underperforms on subtle defects with low contrast against the background texture. Nevertheless, comparative results show that EAMNet, via its cross-episode adaptive analysis, can capture potential defect cues to coarsely segment the main defect area, outperforming other popular networks in such challenging scenarios.

### Ablation study

We perform ablation studies using the VGG-16 backbone in the 1-shot setting on the Surface Defect-$4^i$ dataset.

#### Components analysis

EAMNet comprises four key components: the Episode Adaptive Memory Unit (EAMU) for cross-episode interaction, the Context Adaptation Module (CAM), Global Response Mask Average Pooling (GRMAP), and Attention Distillation (AD). Table 4 summarizes the effectiveness of each component. As the most critical component for facilitating cross-episode interaction, EAMU contributes a significant $5.69\%$ improvement in mIoU. Meanwhile, CAM, GRMAP, and AD are also essential. Collectively, these four modules enable EAMNet to achieve state-of-the-art performance.

**Table 4:** Ablation studies of each component on Surface Defect-$4^i$.

Components				1-shot				$\Delta$
$\rho$	$\psi$	$\boldsymbol{P}_\textrm{supp}$	$\mathscr {L}_\textrm{AD}$	Fold-0	Fold-1	Fold2	Mean	
$\checkmark$				34.88	30.24	21.19	28.77	$\uparrow$5.69
$\checkmark$	$\checkmark$			35.64	31.57	22.16	29.79	$\uparrow$6.71
$\checkmark$	$\checkmark$	$\checkmark$		45.77	31.24	24.78	33.93	$\uparrow$10.85
$\checkmark$	$\checkmark$	$\checkmark$	$\checkmark$	**47.01**	**32.91**	**25.46**	**35.13**	$\uparrow$**12.05**
Baseline				28.33	24.54	16.38	23.08	-

#### $\beta$ in CAM

To investigate the performance of our contextual adaptive factor within the CAM, we evaluated $\beta$ values from $\{0.1,0.2,0.3,0.4,0.5\}$. As shown in Table 5, EAMNet achieves its peak performance with $\beta = 0.4$, and the second-best result with $\beta = 0.5$. Consequently, we set $\beta$ to 0.4 for all subsequent experiments.

**Table 5:** 1-shot mIoU of ablation study for hyperparameter $\beta$ on Surface Defect-$4^i$.

$\beta$	1-shot				$\Delta$
	Fold-0	Fold-1	Fold-2	Mean	
0.10	45.30	30.62	24.26	33.40	$\uparrow$10.31
0.20	45.48	30.92	24.20	33.53	$\uparrow$10.45
0.30	46.44	31.47	24.40	34.10	$\uparrow$11.02
0.40	**47.01**	**32.91**	25.46	**35.13**	$\uparrow$**12.05**
0.50	47.00	32.13	**25.57**	34.90	$\uparrow$11.82
Baseline	28.33	24.54	16.38	23.08	–

#### $\alpha$ in loss function

As shown in Table 6, we compare the various hyperparameter $\alpha$ which lies in our loss function to validate the effectiveness of AD. When the $\alpha =0.05$, we can see this strategy achieves $12.05\%$ mIoU improvement and outperforms other settings.

**Table 6:** 1-shot mIoU of ablation study for hyperparameter $\alpha$ on Surface Defect-$4^i$.

$\alpha$	1-shot				$\Delta$
	Fold-0	Fold-1	Fold-2	Mean	
0.01	**47.48**	30.89	25.45	34.61	$\uparrow$11.53
0.03	47.46	31.51	25.25	34.74	$\uparrow$11.66
0.05	47.01	32.91	**25.46**	**35.13**	$\uparrow$**12.05**
0.07	46.81	**33.36**	24.91	35.03	$\uparrow$11.94
0.09	46.87	32.08	24.85	34.60	$\uparrow$11.52
Baseline	28.33	24.54	16.38	23.08	–

## Conclusions

We propose an episode adaptive memory network (EAMNet) with four major parts (i.e., EAMU, CAM, GRMAP and AD) to the few-shot semantic segmentation for metal surface defect. The EAMU generates an adaptive factor at the cross-episode diagram while alleviating the limited adaptability in semantic description of defect regions within the conventional single-episode training stage. The CAM and GRMAP obtains fine-grained resolution from the contextual adaptive factor and support guidance, as a supplement to the semantic information in a single episode. An attention distillation is designed to store the memory of semantic defect cues between episodes and boost adaptive performance by leveraging semantic attention correspondence. Comprehensive experiments show that EAMNet achieves state-of-the-art performance under all settings.

## Data Availability

All data and codes underlying the results of this study are available at the following URL: https://doi.org/10.5281/zenodo.18174740.
